# Lactate dehydrogenase-to-albumin ratio and adverse outcomes in patients with HFrEF and HFmrEF

**DOI:** 10.3389/fcvm.2026.1786253

**Published:** 2026-04-27

**Authors:** Yaping Zhang, Qianyu Ma, Nana Hu, Bingxin Men, Junlan Zhang, Xiaolei Shi, Jin Zhang

**Affiliations:** 1 First Clinical Medical College, Lanzhou University, Lanzhou, Gansu Province, China; 2 Department of Cardiology, First Hospital of Lanzhou University, Lanzhou, Gansu Province, China; 3 Key Laboratory of Cardiovascular Diseases, Lanzhou, Gansu Province, China

**Keywords:** competing risks model, heart failure with mildly reduced ejection fraction, heart failure with reduced ejection fraction, lactate dehydrogenase-to-albumin ratio, prognosis

## Abstract

**Objective:**

This study investigated the association between lactate dehydrogenase-to-albumin ratio (LAR) and adverse outcomes in patients with heart failure with reduced ejection fraction (HFrEF) and heart failure with mildly reduced ejection fraction (HFmrEF).

**Methods:**

This retrospective cohort study included 1,084 hospitalised patients with HFrEF or HFmrEF. It was approved by the Ethics Committee. Associations between LAR and endpoint events(HF-related readmissions, all-cause deaths, composite endpoints) were assessed using Cox regression, Kaplan–Meier curves, and restricted cubic spline analysis. The Fine-Grey model was applied for HF-related readmission to account for competing mortality. Subgroup and sensitivity analyses were performed to evaluate the robustness of the findings. Incremental predictive value of LAR was assessed using C-index, NRI, and IDI.

**Results:**

During a mean follow-up of 29.3 months, higher LAR was independently associated with increased risks of HF-related readmissions(HR:1.602, 95% CI:1.088–2.359), all-cause mortality (HR:2.008, 95% CI:1.155–3.492), and the composite endpoint (HR:1.648, 95% CI:1.184–2.295) (all *P* < 0.05). Kaplan–Meier analysis confirmed the highest cumulative event incidence in the highest LAR tertile (Log-rank *P* < 0.001). Restricted cubic spline analysis revealed a nonlinear relationship between LAR and all-cause mortality, with risk significantly increasing at LAR ≥ 4.82 (*P*-nonlinear < 0.001). The robustness of these findings was supported by multivariate subgroup and sensitivity analyses.

**Conclusion:**

Baseline LAR level is an independent predictor of HF readmission, all-cause mortality, and the combined endpoint in both HFrEF and HFmrEF patients, demonstrating clinical value for risk stratification.

## Graphical Abstract

**Figure:**
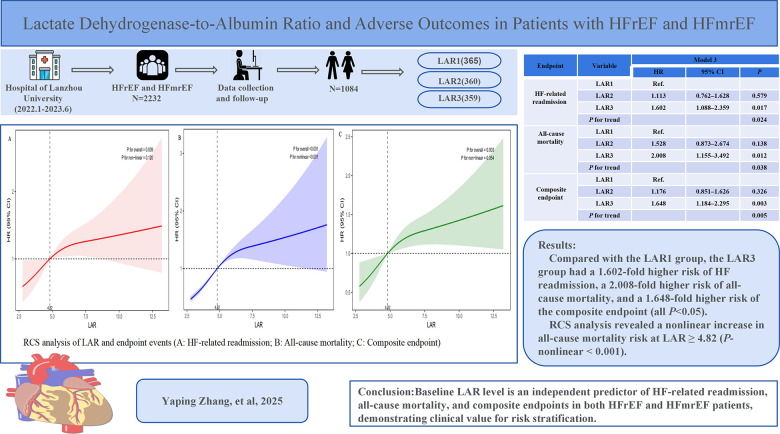
Icons from Pinclipart (https://www.pinclipart.com/): “Hospital Emoji Icon”, “Transparent Group Of People”, “Person Icons Computer” and “Unlabelled Image Of The Anterior View Of The Heart”. “Simple Male and Female Human” icon by Vivek Kumar, licensed under CC BY.

## 1 Introduction

Heart failure (HF) is an end-stage clinical syndrome of all cardiovascular diseases. Despite advances in drug and device therapies, the readmission and mortality rates among HF patients remain high. According to a recent Chinese study, the incidence rates of cardiovascular death or HF rehospitalisation among HF patients at 6, 12, and 24 months post-discharge were 8.9%, 16.1%, and 23.5%, respectively. Furthermore, patients with heart failure with reduced ejection fraction (HFrEF) and heart failure with mildly reduced ejection fraction (HFmrEF) had higher rates of major adverse events than those with heart failure with preserved ejection fraction (HFpEF) (1). Another study reported that the all-cause mortality rates among hospitalised HF patients in China at 30 days, 1 year, and 3 years post-discharge were 2.4%, 13.7%, and 28.2%, respectively (2). Multiple studies have demonstrated that hypoxia, metabolic disorders, and inflammation induce oxidative stress in hypertrophied hearts, exacerbating myocardial inflammatory responses. This leads to myocardial cell apoptosis, myocardial remodelling, and impaired calcium handling capacity, resulting in further deterioration of cardiac function (3–5). Therefore, from the perspective of HF pathogenesis, it is necessary to explore a simple and easily accessible indicator to predict adverse outcomes in patients with HFrEF and HFmrEF.

Lactate dehydrogenase (LDH) is an enzyme widely distributed throughout the human body, participating in the glycolysis process. Albumin (ALB) is a plasma protein synthesised by the liver, involved in numerous physiological processes within the human body. The lactate dehydrogenase to albumin ratio (LAR) is an emerging marker reflecting inflammation, nutritional status, and oxidative stress. Most studies indicate that LAR can predict sepsis-associated acute kidney injury (6, 7), tumour treatment efficacy (8, 9), and all-cause mortality in ischemic stroke (10). However, studies examining LAR in relation to adverse outcomes in patients with HFrEF and HFmrEF remain scarce. This study aims to investigate the relationship between LAR and adverse outcomes in patients with HFrEF and HFmrEF, thereby providing theoretical support for the early identification of high-risk patients, facilitating proactive interventions, and enhancing their prognosis.

## 2 Methods

### 2.1 Study design and participants

**Figure 1:**
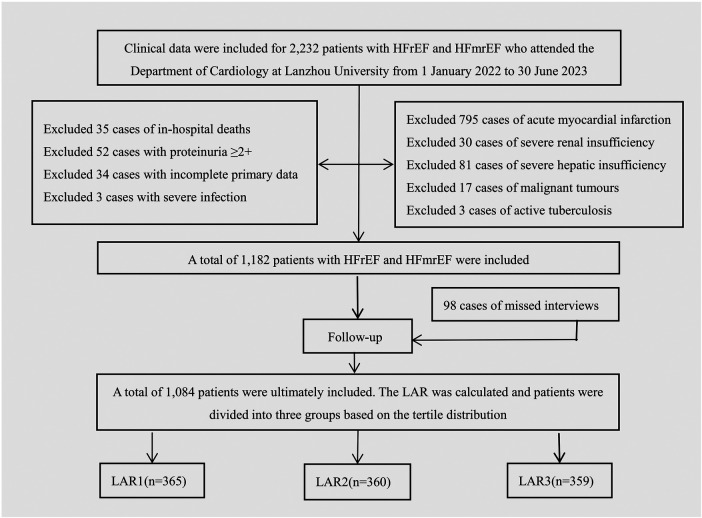
Flowchart depicting the selection of participants.

This study is a retrospective analysis. A total of 2,232 patients with HFrEF and HFmrEF who visited the Department of Cardiology at the First Hospital of Lanzhou University between January 1, 2022, and June 30, 2023, were consecutively enrolled. After screening based on inclusion and exclusion criteria, 1,182 patients were initially included. During follow-up, 98 patients (8.3%) were lost to follow-up due to changes in contact information, unanswered phone calls, or refusal to continue participation, resulting in a final cohort of 1,084 patients. The screening process is illustrated in Figure 1. This study was approved by the Ethics Committee of LZU No.1 Hospital (Ethics Approval No.LDYYLL2025-2101).

Inclusion Criteria: (1) Age 18 years ≤ age < 90 years; (2) Patients meeting the diagnostic criteria for HFrEF or HFmrEF in the Chinese Guidelines for Diagnosis and Treatment of Heart Failure 2024 based on symptoms, signs, laboratory tests, and echocardiography, or patients with a history of HF who had an LVEF ≤ 49% at the time of admission.

Exclusion Criteria: (1) Incomplete primary data. (2) Malignant tumours, severe infectious diseases, active tuberculosis, and autoimmune diseases, as these conditions may elevate LDH levels and reduce albumin, which could confound the study results. (3) Pregnant individuals were excluded from the study as a special population. (4) Severe hepatic or renal insufficiency and significant proteinuria (urine protein ≥ ++) were excluded, as these conditions critically affect drug metabolism and heart failure biomarker levels, thereby introducing major confounding factors. (5) In-hospital deaths were excluded from the analysis due to the study's focus on post-discharge outcomes. (6) We excluded cases of acute myocardial infarction to avoid confounding from the substantial LDH elevation associated with this condition.

### 2.2 Data collection

#### 2.2.1 General information

Record the patient's age, gender, systolic blood pressure, diastolic blood pressure, height, weight, history of hypertension, history of diabetes, history of stroke, prior history of percutaneous coronary intervention (PCI), and comorbidities (including concomitant atrial fibrillation or atrial flutter, ventricular arrhythmias), NYHA functional class, echocardiographic parameters, and post-discharge medication regimen including beta-blockers, angiotensin-converting enzyme inhibitors (ACEI), angiotensin receptor blocker (ARB), angiotensin-neprilysin inhibitor (ARNI), mineralocorticoid receptor antagonist (MRA), sodium-glucose cotransporter 2 inhibitor (SGLT2i), and anticoagulants.

#### 2.2.2 Laboratory tests

Within 24 h of admission, the following parameters were obtained using standard laboratory testing methods: white blood cell count (WBC), haemoglobin concentration (HGB), platelet count (PLT), uric acid (UA), homocysteine (HCY), Glucose (GLU), Creatinine (CR), Troponin I (TNI), N-terminal pro-B-type natriuretic peptide (NT-proBNP), ALB, LDH, D-dimer, Serum sodium, Serum potassium.

#### 2.2.3 Ultrasound in cardiology

Examinations are conducted using the LOGIO9 and PHILIPS IE33 ultrasound diagnostic systems. Each parameter is measured over 3–5 consecutive cardiac cycles, with the average value recorded. The examinee was positioned in the left lateral decubitus position with synchronised electrocardiography connected. The subject was instructed to breathe calmly. Three levels were acquired: parasternal long-axis view of the left ventricle, parasternal short-axis view of the left ventricle (tendon sheath, papillary muscle, and apical levels), and apical two-chamber and apical four-chamber views. Measurements were performed by a trained professional. Parameters included: Left Ventricular Long Diameter systolic (LVLDs), Left Ventricular Long Diameter diastolic (LVLDd), Right Ventricular Anteroposterior Diameter (RVAPD), Left Ventricular Ejection Fraction (LVEF).

### 2.3 Diagnosis criteria

1. HFrEF: Symptoms or signs; LVEF ≤ 40%; HFmrEF: Symptoms or signs; LVEF 41%–49%, according to Chinese guidelines for the diagnosis and treatment of heart failure 2024 (11).
2. Hypertension: Clinic blood pressure ≥140/90 mmHg on three separate occasions without antihypertensive medication; or home blood pressure ≥135/85 mmHg measured over 5–7 consecutive days; or 24 h ambulatory blood pressure ≥130/80 mmHg, with daytime blood pressure ≥135/85 mmHg and nighttime blood pressure ≥120/70 mmHg; or a history of hypertension with current antihypertensive medication use, according to Clinical practice guideline for the management of hypertension in China (12).
3. Diabetes: History of diabetes or currently receiving hypoglycemic medication or insulin therapy; typical diabetes symptoms + random blood glucose ≥11.1 mmol/L or fasting blood glucose ≥7.0 mmol/L or 2 h oral glucose tolerance test ≥11.1 mmol/L or HbA1c ≥ 6.5%; individuals without typical diabetes symptoms require follow-up testing for confirmation, according to Classification and Diagnosis of Diabetes: Standards of Medical Care in Diabetes-2021 (13).
4. Atrial fibrillation: A previously established diagnosis of atrial fibrillation, or a single-lead electrocardiogram (≥30 s) or 12-lead electrocardiogram (≥10 s) showing absent *P* waves, irregular f waves, and absolutely unequal RR intervals, according to 2020 ESC Guidelines for the diagnosis and management of atrial fibrillation developed in collaboration with the European Association for Cardio-Thoracic Surgery (EACTS) (14).
5. Ventricular arrhythmias: including frequent ventricular premature beats (>500/24 h), polymorphic, sustained, torsades de pointes ventricular tachycardia, ventricular flutter, ventricular fibrillation (15).
6. Stroke: A diagnosis of stroke can be made based on a history of previous stroke or neuroimaging evidence of a responsible ischemic lesion, regardless of the duration of symptoms/signs. However, when imaging evidence of a responsible lesion is unavailable, symptoms/signs persisting for more than 24 h remain the threshold. For patients with transient ischemic attacks, symptoms typically last no longer than 0.5–1 h (16).
7. BMI: Calculated as weight (kg) divided by height (m) squared.
8. Estimated glomerular filtration rate (eGFR): Calculated using the formula eGFRcr = 142 * min(Scr/k, 1)^*α* * max(Scr/k, 1)—1.200 * 0.9938 * age * 1.012 [for females]
9. LAR: Calculated based on LDH/ALB.

### 2.4 Subject grouping

Based on the calculation of LAR levels, the quartile groups are as follows: LAR1 ≤ 4.3; 4.3 < LAR2 ≤ 5.53; LAR3 > 5.53.

### 2.5 Follow-up and endpoints

All patients were followed up via telephone, outpatient visits, and readmission records starting from discharge. Follow-up continued until death occurred or July 22, 2025. Endpoint events were defined as: HF-related readmission: The first hospitalisation due to symptoms and signs of heart failure following discharge. All-cause mortality: Total deaths from any cause following discharge. Composite endpoint: HF-related readmission or all-cause mortality.

### 2.6 Statistical methods

Statistical analysis was performed using SPSS version 26.0 and R software version 4.4.2. Variables meeting normal distribution criteria were expressed as mean ± SD, with intergroup differences assessed using one-way analysis of variance (ANOVA). Non-normally distributed variables were presented as median (P25, P75), and comparisons among the three groups were conducted using the Kruskal–Wallis *H* test. Categorical variables were expressed as percentages, with intergroup comparisons performed using chi-square tests or Fisher's exact tests.

Cox proportional hazards model assumptions were tested using Schoenfeld residuals. The global proportional hazards assumption was satisfied for all models across the three endpoints, and the tertiles of LAR also met the proportional hazards assumption (all *P* > 0.05), indicating that all Cox regression models fulfilled the proportional hazards assumption (Supplementary Table S1).

The maximum variance inflation factor for all variables after testing was below 10, indicating no severe multicollinearity (Supplementary Table S2).

Variable Selection: Univariate Cox regression analyses were performed separately for the composite endpoint, all-cause mortality, and readmission for heart failure. Variables with *P* < 0.05 were selected for inclusion in the multivariate Cox regression models for each outcome, taking clinical relevance into account. The results of the univariate analyses for each outcome are detailed in Supplementary Table S3.

Multivariate Cox Regression: For each outcome event, three stepwise models (Models 1–3) were constructed. The adjusting variables for each model are detailed in the corresponding table footnotes.

**Table 2:** Comparison of LAR levels with endpoint events in HFrEF and HFmrEF patients.

Endpoint events	Total (1,084)	LAR1 ≤ 4.3 (365)	4.3 < LAR2 ≤ 5.53 (360)	LAR3 > 5.53 (359)	*P*
HF-related	240 (22.14)	50 (13.70)	72 (20.00)	118 (32.87)^a^^,^^b^	<0.001
readmission [*n*(%)]					
All-cause	142 (13.10)	19 (5.21)	41 (11.39)^a^	82 (22.84)^a^^,^^b^	<0.001
death [*n*(%)]					
Composite	353 (32.56)	66 (18.08)	105 (29.17)^a^	182 (50.70)^a^^,^^b^	<0.001
endpoints [*n*(%)]					

LAR, calculated based on LDH/ALB.

a Compared with LAR1, *P* < 0.05.

b Compared with LAR2, *P* < 0.05.

Multivariate Cox proportional hazards regression models were used to assess the relationship between LAR and endpoint events in patients with HFrEF and HFmrEF. Results were presented as hazard ratios (HR) with 95% confidence intervals (CI). Kaplan–Meier survival curves (K-M) assessed cumulative endpoint incidence across different LAR groups. Restricted cubic splines (RCS) explored potential nonlinear associations between LAR and endpoints. To evaluate potential subgroup differences in LAR's impact on HF-related readmissions, subgroup analyses and interaction tests were conducted based on potential covariates. An incremental Cox regression model was used to assess the predictive value of LAR compared with NT-proBNP, with further assessment using the C-index, NRI, and IDI. In sensitivity analyses, to accurately assess the association between LAR and hospital readmission for heart failure by excluding the competing risk of all-cause mortality, the Fine-Grey competing risks model was employed to calculate the subdistribution hazard ratio (SHR) of LAR on HF rehospitalisation risk and its 95% CI, which were compared with the primary Cox analysis results. Additionally, the association between LAR and endpoint events was further analysed in patients, excluding those who died within <30 days. *P* < 0.05 was considered statistically significant.

## 3 Results

### 3.1 Baseline characteristics of the LAR grouped population

This research included 1,084 patients with HFrEF and HFmrEF. Patients were divided into three groups based on LAR levels: LAR1 ≤ 4.3 (365 cases); 4.3 < LAR2 ≤ 5.53 (360 cases); LAR3 > 5.53 (359 cases). Compared with the LAR1 group, the LAR3 group exhibited significant differences in baseline characteristics (*P* < 0.05), characterised by: higher diastolic blood pressure levels and a greater proportion with concomitant atrial fibrillation or atrial flutter at admission; poorer cardiac function classification, while a lower proportion had a history of PCI. Laboratory test results showed lower levels of WBC, HGB, ALB, and eGFR, while LDH, CR, UA, HCY, TNI, NT-proBNP, D-dimer, and LAR levels were all higher. Concurrently, echocardiographic measurements showed higher LAD and RVAPD, while LVEF was lower. Among patients discharged with medication orders, the LAR3 group exhibited significantly higher proportions of MRA, SGLT2i, and anticoagulant use compared to the LAR1 group (*P* *<* *0.05)*. However, no significant differences were observed in BMI, hypertension, diabetes, history of stroke, concomitant ventricular arrhythmia, PLT, GLU, serum sodium, serum potassium, LVLDs, LVLDd, β-blockers, or ACEI/ARB/ARNI use (*P* > 0.05). Detailed results are shown in Table 1.

**Table 1:** Baseline characteristics of the LAR-grouped population.

Variable	Total population	LAR1 ≤ 4.3	4.3 < LAR2 ≤ 5.53	LAR3 > 5.53	*P value*
	(1,084)	(365)	(360)	(359)	
Basic information					
Age [years, M(Q1, Q3)]	62.00 (54.75, 70.00)	59.00 (52.00, 66.50)	64.00 (55.00, 71.00)	64.00 (55.00, 72.00)	<0.001
Gender [*n*(%)]					<0.001
Male	853 (78.69)	309 (84.66)	286 (79.44)	258 (71.87)^a^	
Female	231 (21.31)	56 (15.34)	74 (20.56)	101 (28.13)^a^	
Admission systolic blood pressure	123.00 (109.00, 138.00)	120.00 (109.00, 135.00)	126.00 (110.00, 141.00)	122 (106.00, 139.00)	0.009
[mmHg, M(Q1, Q3)]					
Admission diastolic blood pressure	78.00 (68.00, 89.00)	76.00 (68.00, 86.00)	80.00 (69.00, 90.00)	80.00 (69.00, 91.00)	0.021
[mmHg, M(Q1, Q3)]					
BMI{[kg/m^2^, M(Q1, Q3)]}	24.41 (22.04, 26.78)	24.49 (22.77, 26.78)	24.22 (21.97, 26.71)	24.41 (21.60, 26.93)	0.442
Past medical history and comorbidities					
History of hypertension [*n*(%)]	486 (44.83)	151 (41.37)	166 (46.11)	169 (47.08)	0.254
History of diabetes [*n*(%)]	256 (23.62)	94 (25.75)	72 (20)	90 (25.07)	0.138
Previous history of PCI [*n*(%)]	398 (36.72)	179 (49.04)	136 (37.78)^a^	83 (23.12)^a,b^	<0.001
History of stroke [*n*(%)]	64 (5.90)	19 (5.21)	19 (5.28)	26 (7.24)	0.421
Combined with atrial fibrillation and atrial flutter [*n*(%)]	237 (21.86)	38 (10.41)	75 (20.83)^a^	124 (34.54)^a,b^	<0.001
Combined with ventricular arrhythmia [*n*(%)]	289 (26.66)	88 (24.11)	92 (25.56)	109 (30.36)	0.138
Cardiac function classification (NYHA) [*n*(%)]					<0.001
Class II	403 (37.18)	179 (41.10)	132 (30.28)^a^	92 (23.96)^a,b^	
Class III	435 (40.13)	134 (36.71)	154 (42.78)	147 (40.95)	
Class IV	246 (22.69)	52 (14.25)	74 (20.56)	120 (33.43)^a,b^	
Laboratory tests					
WBC [×10^9^/L, M(Q1, Q3)]	6.25 (5.15, 7.51)	6.02 (5.11, 7.47)	6.20 (5.08, 7.24)	6.57 (5.26, 7.97)	0.041
HGB [g/L, M(Q1, Q3)]	151.00 (137.00, 163.00)	152.00 (141.00,163.00)	150.50 (136.75,164.00)	149.00 (132.00,163.00)^a^	0.049
PLT [×10^9^/L, M(Q1, Q3)]	181.00 (143.00, 221.00)	185.00 (148.50, 219.50)	177.00 (140.25, 219.00)	180.00 (137.25, 223.00)	0.377
GLU [mmol/L, M(Q1, Q3)]	5.82 (4.91, 7.67)	5.80 (4.82, 7.50)	5.75 (4.87, 7.56)	5.95 (5.02, 8.00)	0.154
ALB [g/L, M(Q1, Q3)]	42.70 (39.68, 45.00)	44.40 (42.55, 46.50)	43.00 (40.40, 45.08)	39.30 (36.50, 42.30)	<0.001
LDH [u/L, M(Q1, Q3)]	205.00 (177.00, 242.25)	165.00 (150.00, 179.00)	205.00 (194.00, 221.00)	265.00 (240.00, 306.00)	<0.001
CR [umol/L, M(Q1, Q3)]	81.60 (69.88, 95.93)	77.80 (67.85, 89.85)	81.00 (69.70, 94.00)	87.2 (74.10, 103.80)	<0.001
eGFR [mL/min/1.73 m², M(Q1, Q3)]	92.77 (76.36, 101.84)	97.31 (83.73, 104.27)	92.13 (78.52, 100.54)	84.07 (68.80, 98.98)	<0.001
Blood sodium [mmol/L, M(Q1, Q3)]	139.70 (137.97, 141.20)	139.70 (138.00, 141.05)	140.00 (138.43, 141.58)	139.20 (136.60, 141.20)	0.001
Blood potassiu [mmol/L, M(Q1, Q3)]	3.98 (3.72, 4.26)	3.98 (3.75, 4.24)	4.02 (3.74, 4.28)	3.96 (3.67, 4.27)	0.455
UA [umol/L, M(Q1, Q3)]	367.00 (302.75, 457.00)	354.00 (290.50, 426.00)	353.00 (293.25, 430.50)	407.00 (326.00, 524.00)	<0.001
HCY [umol/L, M(Q1, Q3)]	19.90 (15.00, 27.50)	18.90 (14.40, 25.50)	20.20 (15.70, 29.78)	20.90 (15.30, 27.40)	0.013
TNI [ng/mL, M(Q1, Q3)]	0.01 (0.01, 0.01)	0.01 (0.01, 0.01)	0.01 (0.01, 0.01)	0.01 (0.01, 0.03)	<0.001
NT-ProBNP [pg/mL, M(Q1, Q3)]	1,705.00 (583.50, 4,470.00	769.00 (284.00, 1,690.00)	1,720.00 (614.50, 3,627.50)	4,140.00 (1,720.00, 8,940.00)	<0.001
D-dimer [ug/mL, M(Q1, Q3)]	0.41 (0.24, 0.91)	0.27 (0.18, 0.43)	0.40 (0.24, 0.74)	0.87 (0.41, 1.77)	<0.001
LAR [M(Q1, Q3)]	4.82 (4.01, 5.94)	3.75 (3.43, 4.02)	4.83 (4.55, 5.16)	6.59 (5.94, 7.84)	<0.001
Echocardiography					
LAD [cm, M(Q1, Q3)]	3.90 (3.60, 4.40)	3.80 (3.40, 4.20)	3.90 (3.60, 4.30)	4.20 (3.80, 4.60)	<0.001
LVLDs [cm, M(Q1, Q3)]	7.10 (6.40, 7.70)	7.10 (6.50, 7.70)	7.00 (6.33, 7.60)	7.00 (6.40, 7.80)	0.168
Echocardiography					
LVLDd [cm, M(Q1, Q3)]	7.90 (7.30, 8.60)	8.00 (7.50, 8.60)	7.80 (7.30, 8.50)	7.90 (7.30, 8.60)	0.046
RVAPD [cm, M(Q1, Q3)]	2.30 (2.08, 2.50)	2.20 (2.00, 2.40)	2.30 (2.00, 2.40)	2.40 (2.20, 2.80)	<0.001
LVEF [%, M(Q1, Q3)]	41.00 (35.00, 46.00)	43.00 (37.00, 47.00)	42.00 (36.00, 46.00)	37.00 (32.00, 43.00)	<0.001
Discharge doctor's instructions for medication					
Beta-blockers [*n*(%)]	978 (90.22)	337 (92.33)	322 (89.44)	319 (88.86)	0.242
ACEI/ARB/ARNI [*n*(%)]	952 (87.82)	319 (87.40)	316 (87.78)	317 (88.30)	0.933
MRA [*n*(%)]	766 (70.66)	207 (56.71)	248 (68.89)	311 (86.63)	<0.001
SGLT2i [*n*(%)]	715 (65.96)	224 (61.37)	229 (63.61)	262 (72.98)	0.002
Anticoagulants [*n*(%)]	259 (23.89)	40 (10.96)	82 (22.78)	137 (38.16)	<0.001

AF, atrial fibrillation; AFL, atrial flutter; BMI, body mass index; WBC, white blood cell count; HGB, hemoglobin; PLT, platelet count; GLU, blood glucose; ALB, albumin; LDH, lactate dehydrogenase; CR, serum creatinine; eGFR, estimated glomerular filtration rate; UA, uric acid; HCY, homocysteine; Troponin I, TNI; NT-proBNP, N-terminal pro-B-type natriuretic peptide; LDH/ALB ratio, lactate dehydrogenase to albumin ratio; LA diameter, left atrial diameter; LVSD, left ventricular systolic diameter; LVSDd, left ventricular diastolic diameter; RVAPD, right ventricular anterior-posterior diameter; LVEF, left ventricular ejection fraction; ACEI, angiotensin-converting enzyme inhibitor; ARB, angiotensin receptor blocker; ARNI, angiotensin neuropeptidase inhibitor; MRA, mineralocorticoid receptor antagonist; SGLT2i, sodium-glucose cotransporter 2 inhibitor. (1 mmHg = 0.133322 kPa).

a Compared with LAR1, *P* < 0.05.

b Compared with LAR2, *P* < 0.05.

Notably, owing to the large sample size, most variables with statistical significance showed minimal absolute between-group differences and were not clinically meaningful. Only a few indicators reflecting disease severity demonstrated relatively obvious differences, which represented a consistent clinical gradient corresponding to LAR grouping rather than baseline imbalance.

### 3.2 Association between LAR levels and endpoint events in patients with HFrEF and HFmrEF

With a mean follow-up of 29.3 months, a total of 240 HF-related readmissions (22.14%), 142 all-cause deaths (13.10%), and 353 composite endpoints (32.56%) were recorded. Patients in the LAR3 group demonstrated significantly higher rates of Cardiac failure readmission, all-cause mortality, and Compound endpoint occurrence compared to both the LAR1 and LAR2 groups (*P* < 0.001). As shown in Table 2.

### 3.3 Cox regression analysis of LAR with HFrEF and HFmrEF endpoint events

Multivariate Cox proportional hazards regression analyses were performed to assess the association between the LAR and endpoint events. Variables with *P* < 0.05 in the univariate Cox analysis were included in the multivariate Cox regression model for further analysis. Three Cox regression models were constructed to explore the independent association between LAR and the unfavourable outcome. In Model 3, the risk of re-admissions for heart failure in the LAR3 group was 1.602 times that of the LAR1 group (HR:1.602, 95% CI: 1.088–2.359, *P* = 0.017), all-cause mortality risk was 2.008 times that of the LAR1 group (HR:2.008, 95% CI: 1.155–3.492, *P* = 0.012), and the composite endpoint risk was 1.648 times that of the LAR1 group (HR: 1.648, 95% CI: 1.184–2.295, *P* = 0.003). As shown in Table 3.

**Table 3:** Cox regression models for endpoint events in HFrEF and HFmrEF patients.

	Variable	Model 1			Model 2			Model 3		
		HR	95% CI	*P*	HR	95% CI	*P*	HR	95% CI	*P*
HF-related readmission	LAR1	Ref.			Ref.			Ref.		
	LAR2	1.533	1.069–2.199	0.020	1.188	0.882–1.717	0.359	1.113	0.762–1.628	0.579
	LAR3	3.080	2.212–4.288	<0.001	2.220	1.572–3.134	<0.001	1.602	1.088–2.359	0.017
	*P* for trend			<0.001			<0.001			0.024
All-cause mortality	LAR1	Ref.			Ref.			Ref.		
	LAR2	2.260	1.312–3.894	0.003	1.911	1.104–3.307	0.021	1.528	0.873–2.674	0.138
	LAR3	4.843	2.940–7.977	<0.001	3.799	2.278–6.337	<0.001	2.008	1.155–3.492	0.012
	*P* for trend			<0.001			<0.001			0.038
Composite endpoint	LAR1	Ref.			Ref.			Ref.		
	LAR2	1.697	1.247–2.309	0.001	1.413	1.033–1.932	0.031	1.176	0.851–1.626	0.326
	LAR3	3.562	2.687–4.722	<0.001	2.748	2.045–3.694	<0.001	1.648	1.184–2.295	0.003
	*P* for trend			<0.001			<0.001			0.005

Model 1 for all endpoint events was unadjusted.

HF-related readmission: Model 2: Adjusted for age, sex, history of PCI, concomitant atrial fibrillation or atrial flutter, and concomitant ventricular arrhythmia; Model 3: Model 3 was built upon Model 2 by additionally adjusting for the following variables, including admission systolic blood pressure, diastolic blood pressure, HGB, CR, eGFR, serum sodium, UA, TNI, NT-proBNP, D-dimer, LAD, LVLDs, RVAPD, LVEF, MRA, SGLT2i, anticoagulants, and heart function classification.

All-cause mortality: Model 2: Adjusted for age, history of stroke, concomitant atrial fibrillation or atrial flutter, concomitant ventricular arrhythmia,; Model 3:Model 3 was built upon Model 2 by additionally adjusting for the following variables, including WBC, HGB, PLT, CR, eGFR, serum sodium, UA, HCY, TNI, NT-proBNP, D-dimer, LAD, RVAPD, LVEF, MRA, SGLT2i, anticoagulants, and cardiac function classification.

Composite Endpoint: Model 2: Adjusted for age, sex, history of stroke, prior PCI, concomitant atrial fibrillation or atrial flutter, and concomitant ventricular arrhythmia; Model 3: Model 3 was built upon Model 2 by additionally adjusting for the following variables, including admission systolic blood pressure, diastolic blood pressure, HGB, PLT, CR, eGFR, serum sodium, UA, TNI, NT-proBNP, D-dimer, LAD, LVLDs, RVAPD, LVEF, MRA, SGLT2i, anticoagulants, and cardiac function classification.

### 3.4 KM survival analysis of LAR and endpoint events

**Figure 2:**
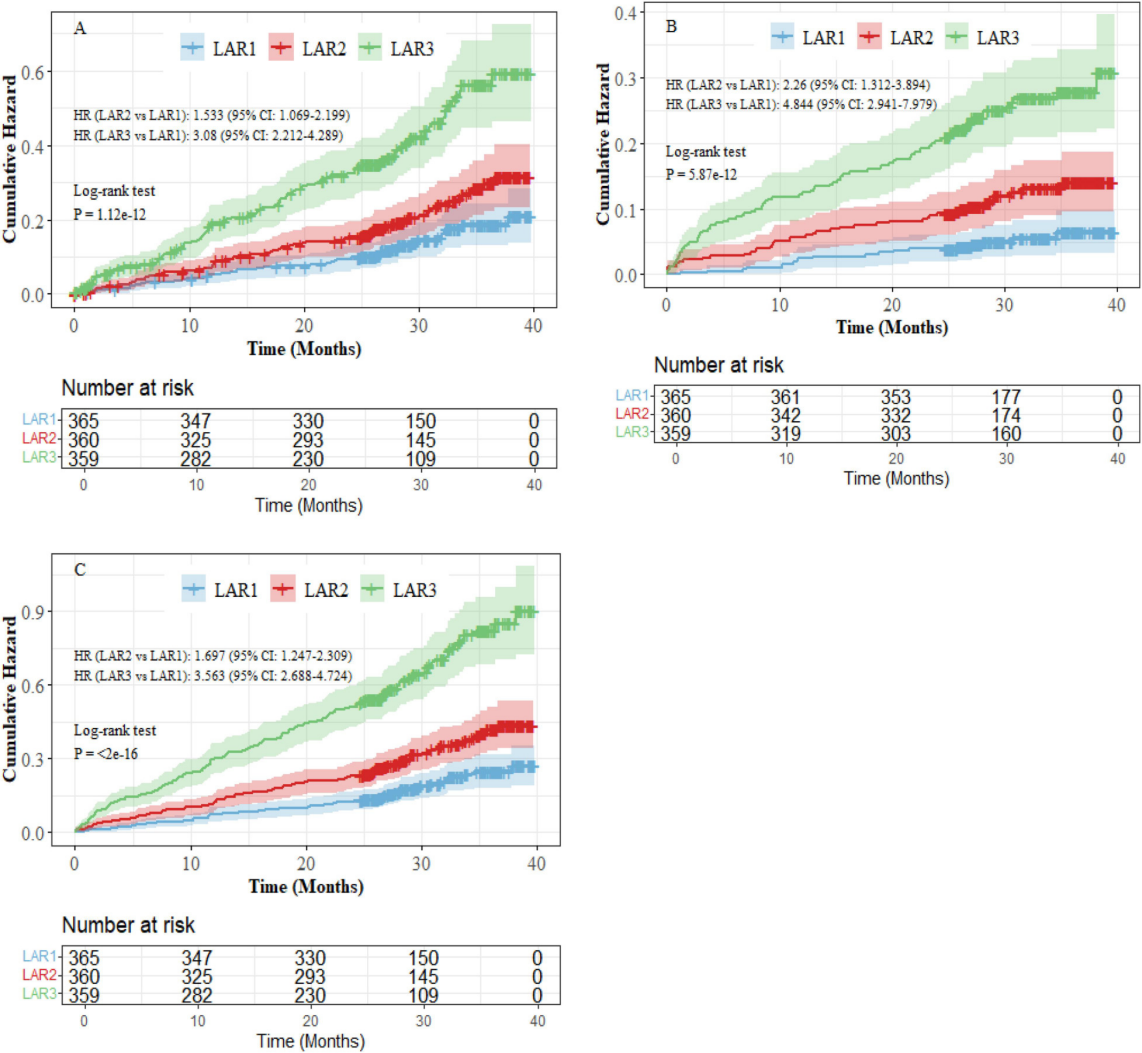
KM survival analysis of LAR and endpoint events. **(A)** HF-related readmission. **(B)** All cause death. **(C)** Composite endpoint.

After a median follow-up of 29.3 months, the risks of HF-related readmission, all-cause mortality, and the composite endpoint progressively increased over time across the study population. Compared with the LAR1 and LAR2 groups, the LAR3 group exhibited significantly higher cumulative risks for these outcomes, with statistically significant differences between groups (Log-rank test, *P* < 0.05 for all comparisons). As shown in Figure 2.

### 3.5 RCS analysis of LAR and terminal events

**Figure 3:**
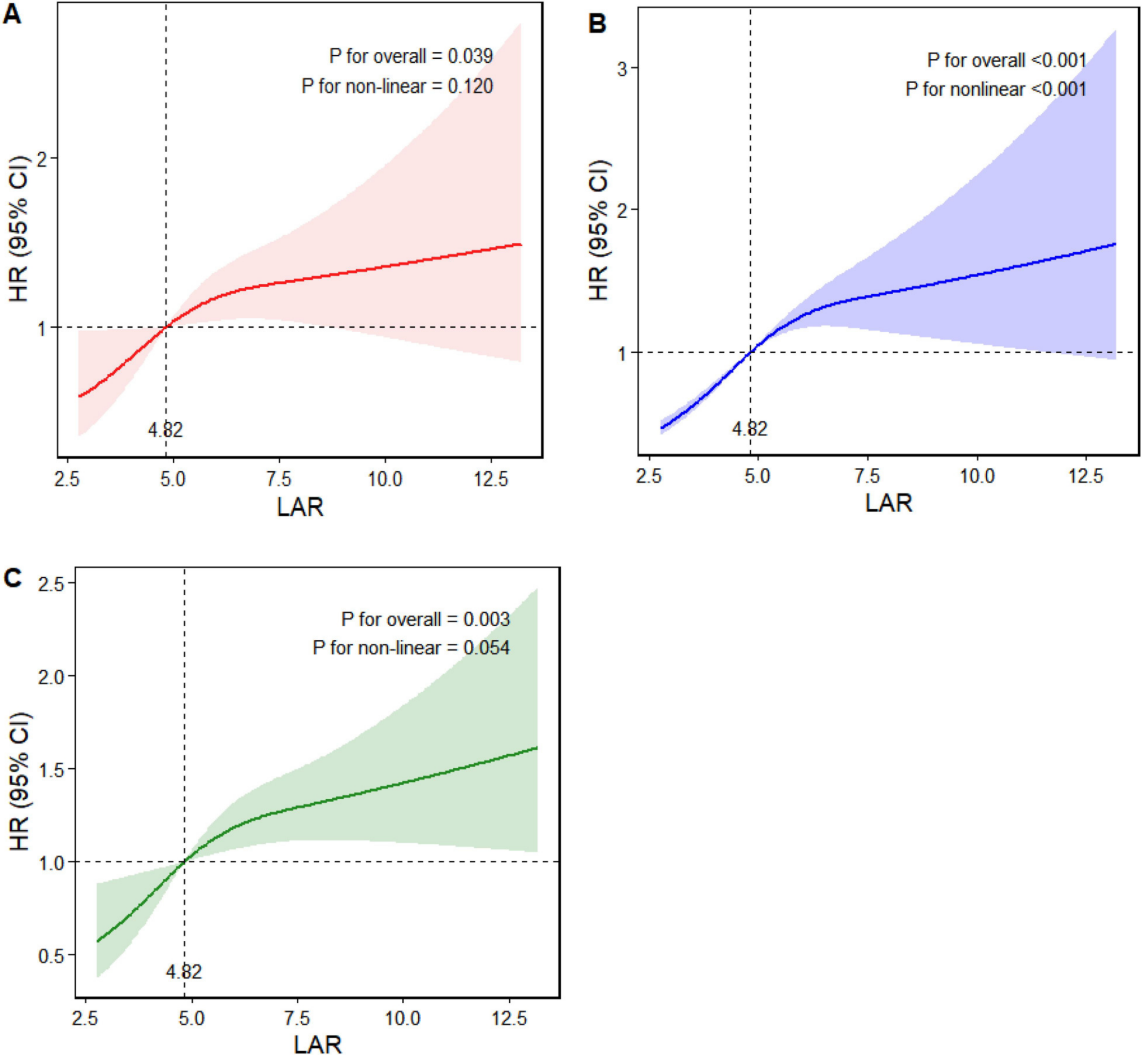
RCS analysis of LAR and endpoint events. **(A)** HF-related readmission. **(B)** All cause death. **(C)** Composite endpoint.

RCS analysis demonstrated that LAR exhibited a linear relationship with HF-related readmission and composite endpoints (*P* for nonlinear = 0.120 and 0.054, respectively), indicating that as LAR levels increased, the risk of these events correspondingly rose. Conversely, LAR exhibited a nonlinear relationship with all-cause mortality (*P* for nonlinear < 0.001) and demonstrated a threshold effect. Specifically, when LAR levels reached ≥4.82, the all-cause mortality rate progressively increased with further rises in LAR levels. Results are presented in Figure 3.

### 3.6 Multivariate subgroup analysis of LAR and HF-related readmission

**Figure 4:**
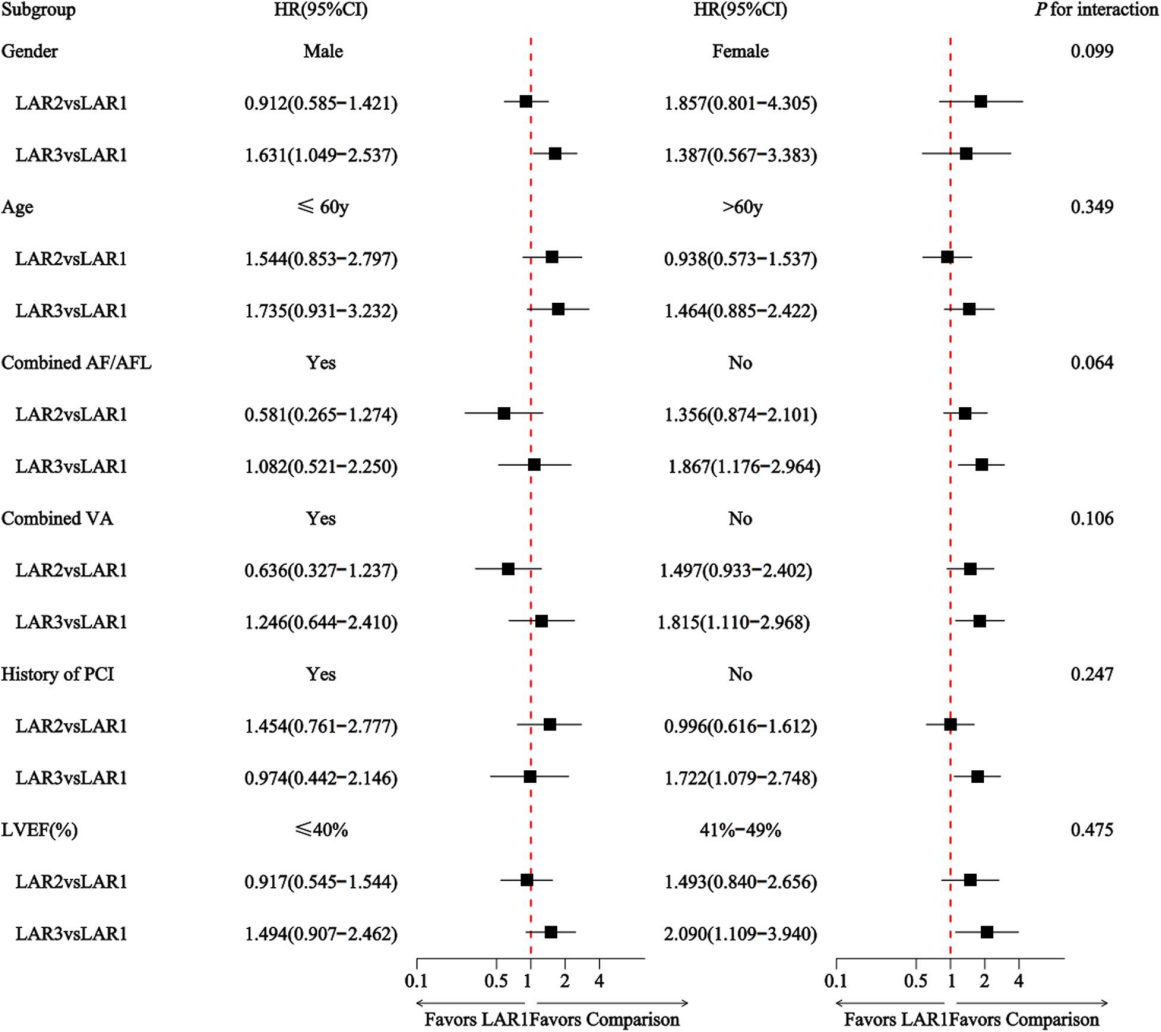
Multivariate subgroup analysis of LAR and HF-related readmission.

Subgroup analyses were performed to evaluate the association between LAR and HF-related readmission across subgroups defined by age, sex, atrial fibrillation/flutter, ventricular arrhythmia, history of PCI, and LVEF. No interaction between subgroups and LAR was observed in subgroup analyses (all interaction *P* values > 0.05). As shown in Figure 4.

### 3.7 Comparison of the predictive value of LAR and NT-proBNP (24 months)

An incremental Cox regression model was used to assess the predictive value of LAR compared with NT-proBNP. Model 1 included age, sex, NYHA functional class, LVEF, eGFR, and log(TnI + 0.001). When NT-proBNP was added to Model 1, it significantly improved the model performance for all three endpoints (ΔC-index 0.020–0.041, all *P* < 0.01). When LAR was added to the NT-proBNP model, the identification of low-risk patients for HF readmission and the composite endpoint was significantly improved (NRI- 0.268 and 0.251, respectively, with 95% CI not crossing 0 in both cases), whilst no significant increment was observed for the all-cause mortality endpoint. As shown in Table 4. Detailed results are available in Supplementary Table S4.

**Table 4:** Comparison of the predictive value of LAR and NT-proBNP (24 months).

Endpoint	Model	C-index (95%CI)	ΔC- index	*P*	NRI (95% CI)	*P*	NRI + (95% CI)	NRI- (95% CI)
HF-relatd readmission	Model 1	0.664 (0.628–0.701)	**-**	**-**	**-**	**-**	**-**	**-**
	+NT-proBNP	0.684 (0.650–0.719)	+0.020	**0**.**007**	**0.227 (0.064–0.393)**	**0.007**	**0.158 (0.036–0.303)**	0.069 (−0.004–0.126)
	+LAR	0.689 (0.655–0.723)	+0.005	0.083	0.171(−0.027–0.381)	0.075	−0.097(−0.193–0.083)	**0.268 (0.114–0.369)**
All-cause mortality	Model 1	0.739 (0.698–0.779)	-	-	-	-	-	-
	+NT-proBNP	0.780 (0.744–0.815)	**+0.041**	0.003	0.426 (0.228–0.650)	**0.004**	**0.232 (0.099–0.397)**	0.193 (0.093–0.278)
	+LAR	0.781 (0.745–0.816)	+0.001	0.733	0.217(−0.116–0.386)	0.131	−0.089(−0.292–0.087)	0.307 (−0.005–0.422)
Composite endpoints	Model 1	0.689 (0.660–0.718)	-	-	-	-	-	-
	+NT-proBNP	0.717 (0.690–0.745)	**+0.029**	0.0001	**0.305 (0.181–0.463)**	**0.004**	**0.176 (0.083–0.292)**	0.129 (0.066–0.200)
	+LAR	0.721 (0.695–0.748)	**+0.004**	0.097	0.167(−0.037–0.333)	0.131	−0.084(−0.193–0.036)	**0.251 (0.112–0.348)**

Model 1: age, sex, NYHA functional class, LVEF, eGFR, log(TnI + 0.001).

NRI, net risk improvement; NRI+, net risk improvement in the event group; NRI–, net risk improvement in the non-event group.

Bold text indicates that the 95% CI does not cross zero, indicating statistical significance.

### 3.8 Sensitivity analysis of LAR and final event

We employ the Fine-Grey competing risks model to assess the more precise relationship between LAR and HF-related readmission. Results indicate that after controlling for the same set of covariates and accounting for competing risks of all-cause mortality, the association between LAR and HF-related readmission remains significant (LAR3 vs. LAR1: SHR = 1.520, 95% CI: 1.030–2.260, *P* = 0.036), consistent with the primary analysis findings. As shown in Table 5.

**Table 5:** Competitive risk analysis of LAR and HF-related readmission and comparison with primary analysis results.

	Variable	Primary analysis: Cox model 3			Sensitivity analysis: competing risks model		
		HR	95% CI	*P*	SHR	95% CI	*P*
HF-related readmission	LAR1	Ref.			Ref.		
	LAR2	1.113	0.762–1.628	0.579	1.100	0.750–1.610	0.640
	LAR3	1.602	1.088–2.359	0.017	1.520	1.030–2.260	0.036
	*P* for trend			0.024			0.026

Cox model 3 and competing risks model: adjusting for the following variables, including age, sex, history of PCI, concomitant atrial fibrillation or atrial flutter, and concomitant ventricular arrhythmia, admission systolic blood pressure, diastolic blood pressure, HGB, CR, eGFR, serum sodium, UA, TNI, NT-proBNP, D-dimer, LAD, LVLDs, RVAPD, LVEF, MRA, SGLT2i, anticoagulants, and heart function classification.

After excluding patients who died within 30 days, the results showed that the associations between LAR and all endpoint events remained significant, further validating the stability of Cox Model 3. As shown in Table 6.

**Table 6:** Cox regression model of LAR and endpoint events (excluding patients with death within <30 days).

	Variables	Model 1			Model 2			Model 3		
		HR	95% CI	*P*	HR	95% CI	*P*	HR	95% CI	*P*
HF-related readmission	LAR1	Ref.			Ref.			Ref.		
	LAR2	1.534	1.069–2.200	0.020	1.288	0.891–1.860	0.178	1.111	0.760–1.625	0.587
	LAR3	3.058	2.195–4.260	<0.001	2.352	1.660–3.332	<0.001	1.589	1.079–2.342	0.019
	*P* for trend			<0.001			<0.001			0.027
All-cause mortality	LAR1	Ref.			Ref.			Ref.		
	LAR2	1.934	1.106–3.381	0.021	1.650	0.940–2.898	0.081	1.290	0.726–2.293	0.385
	LAR3	4.357	2.630–7.218	<0.001	3.444	2.050–5.783	<0.001	1.931	1.099–3.392	0.022
	*P* for trend			<0.001			<0.001			0.042
Composite endpoints	LAR1	Ref.			Ref.			Ref.		
	LAR2	1.603	1.174–2.189	0.003	1.334	0.972–1.832	0.075	1.106	0.797–1.536	0.546
	LAR3	3.420	2.575–4.542	<0.001	2.638	1.958–3.554	<0.001	1.645	1.178–2.298	0.003
	*P* for trend			<0.001			<0.001			0.003

see Table 3.

## 4 Discussion

This study explored the association between LAR and adverse outcomes in populations with HFrEF and HFmrEF. Results showed that after adjusting for multiple confounding variables in Cox regression and RCS analysis, LAR was significantly associated with HF-related rehospitalisation, all-cause mortality, and composite event rates. After adjusting for covariates using a competing risks model to account for the competing effects of all-cause mortality and recurrence of HF, the results remained consistent with the primary Cox regression analysis. Concurrently, Kaplan–Meier survival curves demonstrated significantly higher cumulative risks of HF-related readmission, all-cause mortality, and composite events in the LAR3 group compared to the LAR1 and LAR2 groups. Furthermore, threshold effect analysis revealed an inflexion point of 4.82 for LAR in all-cause mortality, indicating increased all-cause mortality risk above this threshold. This study suggests that LAR serves as an effective risk stratification biomarker for follow-up management in patients with HFrEF and HFmrEF.

Heart failure (HF) represents one of the major public health challenges currently facing China due to its high incidence, mortality, and readmission rates. A study from the China Hypertension Survey found that the prevalence of HF among adults aged ≥35 years was 1.3%, equating to approximately 13.7 million patients (17). A prospective study from the China Heart Failure Registry, encompassing 230,637 patients, demonstrated significant prognostic differences among different heart failure subtypes. The 3-year mortality rate was 31.9% for HFrEF patients and 27.6% for HFmrEF patients, both higher than the corresponding mortality rate for HFpEF patients (2). Survey data indicate that in China, the annual per capita hospitalisation cost for urban heart failure patients is $4,406.8, with outpatient costs at $892.3. A substantial 40.5% of patients experience more than three hospitalisations annually (18). This study demonstrates that after more than two years of follow-up, HFrEF and HFmrEF patients had an all-cause mortality rate of 13.1% and a HF readmission rate of 22.14%, indicating the long-term risks faced by these patient groups.

Studies have confirmed that inflammation, oxidative stress, and nutrition are closely associated with the pathogenesis of HF (19, 20). The DAPA-HF study found that elevated levels of IL-6 and hs-CRP were both associated with an increased risk of HF worsening or cardiovascular death (21). Brendan et al. (22) demonstrated that treatment with the interleukin-1β receptor antagonist canakinumab reduced HF rehospitalisation and HF-related mortality in high-risk patients with a history of myocardial infarction and persistent subclinical inflammation. LDH participates in glucose metabolism by catalysing the conversion between lactate and pyruvate. When tissue cells are damaged, LDH is released into the peripheral blood, and total LDH levels reflect the extent of multi-organ injury. Multiple studies indicate that elevated LDH levels correlate with HF severity, left ventricular remodelling following acute myocardial infarction, and reduced survival rates (23–25). ALB serves as an indicator of human nutritional status, exerting anti-inflammatory, antioxidant, immune-modulating, and colloid osmotic pressure-maintaining effects. HF-induced systemic congestion contributes to hypoalbuminemia by impairing hepatic ALB synthesis, restricting nutritional intake, and exacerbating inflammatory catabolism. This hypoalbuminemia further reduces plasma colloid osmotic pressure, intensifying fluid retention and creating a vicious cycle (26). Tan et al. (27) found that chronic HF patients with ALB ≤ 35 g/L exhibited significantly higher 1-, 3-, and 5-year mortality rates compared to those with ALB > 35 g/L. Although LDH and ALB have been individually demonstrated to correlate with poor HF outcomes, their separate application reflects only one aspect—cellular injury or nutritional-inflammatory status, respectively. LAR, capable of integrating both pathophysiological aspects, may offer superiority in the complex clinical syndrome of HF.

Research indicates that LAR serves as a comprehensive indicator reflecting inflammation, oxidative stress, and nutritional status (28). Studies demonstrate that LAR is significantly associated with in-hospital mortality in patients with acute heart failure (OR = 1.09, *P* < 0.001), with the mean LAR ratio being higher in the mortality group than in the survival group (17.38 vs. 13.44) (29). Lili Ye et al. (30) enrolled 520 patients who experienced cardiac arrest during their first ICU admission. After a 30-day follow-up, the deceased group exhibited significantly higher LDH and LAR levels than the survivors, while albumin levels and ICU length of stay were significantly lower (*P* < 0.05). After adjusting for multiple confounders, LAR ≥ 15.5 remained an independent risk factor for both in-hospital and 30-day mortality among ICU patients. Similarly, this study also identified LAR as a risk factor for poor HF outcomes and a risk stratification indicator. Higher LAR levels were associated with increased risks of HF-related readmission, all-cause mortality, and composite events. However, the threshold for LAR and all-cause mortality in this study was 4.82, lower than that in the aforementioned study. This discrepancy may be attributed to the inclusion of critically ill patients who experienced the malignant event of cardiac arrest in the earlier study. Some patients underwent cardiopulmonary resuscitation and electrical cardioversion, resulting in more severe cellular damage, inflammatory responses, and poorer nutritional status, thus yielding a higher LAR threshold than in this study.

In recent years, other composite indices based on inflammation and albumin have also attracted increasing attention in the prognostic assessment of heart failure. Both the C-reactive protein-to-albumin ratio (CAR) and the Endothelial Activation and Stress Index (EASIX) have been shown to predict short- and long-term mortality in patients with HFrEF (31, 32). CAR and LAR share a similar rationale, both reflecting an inflammatory-nutritional imbalance. EASIX, on the other hand, comprises LDH, platelets and creatinine, reflecting renal dysfunction, cellular turnover and hypoxic injury, and immune-thrombotic imbalance, respectively (33). Although both indices share LDH as a component, EASIX focuses on endothelial dysfunction, whilst LAR is more indicative of cellular metabolic and nutritional imbalance. These three indices reflect the pathophysiological mechanisms of heart failure from different perspectives—namely, inflammation, endothelial function, cellular metabolism and nutritional status—and may complement one another in clinical applications. However, with regard to LAR, the current evidence remains somewhat limited.

Although existing research suggests that LAR may have prognostic value in acute and severe cardiovascular events, the evidence is largely concentrated on short-term and in-hospital mortality. Currently, there remains insufficient robust evidence regarding the impact of LAR on mid-to-long-term outcomes (such as HF-related readmissions) in patients with HFrEF and HFmrEF, or whether this association persists after excluding competing mortality events. This study confirms that LAR is significantly associated with medium to long-term adverse prognosis in HFmrEF and HFrEF, providing a basis for risk stratification and management in this population.

Subgroup analysis results from this study indicate that among patients without concomitant atrial fibrillation/atrial flutter or ventricular arrhythmia during hospitalisation, and without prior PCI history, the LAR3 group exhibited a significantly elevated risk of HF-related readmission. This may be attributed to patients with concomitant AF/AFL or ventricular arrhythmias undergoing catheter ablation and standardised drug consolidation therapy during hospitalisation, which may help mitigate systemic inflammatory responses and thereby improve clinical outcomes. Conversely, in patients with prior PCI history, revascularisation improved myocardial ischemia. Concurrent use of anticoagulants, statins, and drugs inhibiting ventricular remodelling likely exerted synergistic effects in mitigating cellular injury and suppressing inflammation, thereby reducing HF readmission risk. Furthermore, LAR demonstrated the strongest predictive power for risk in HFmrEF patients with LVEF 41%–49% (HR = 2.090), suggesting that the pathological process represented by LAR may be particularly significant in this specific stage of HF. This finding may offer new perspectives for precision management of HFmrEF. It is important to emphasise that the above interpretations represent reasonable extrapolations based on existing data. Despite these trends, the core finding of non-significant interaction remains the most robust conclusion, strongly supporting LAR's potential as a universal risk stratification tool for both HFrEF and HFmrEF patients. These subgroup findings should therefore be viewed as exploratory and hypothesis-generating, rather than confirmatory. Accordingly, beyond the robustness of these associations, the practical implementation of LAR in clinical settings also depends on a well-defined risk stratification strategy.

In this study, patients were stratified into tertiles based on baseline LAR levels. This categorization strategy was chosen to ensure balanced sample sizes across groups and to facilitate clinical interpretability. Notably, the mortality threshold identified by restricted cubic spline analysis (LAR ≥ 4.82) was close to the lower boundary of the third tertile (LAR > 5.53). This indicates that tertile-based grouping effectively captures clinically meaningful risk thresholds while preserving statistical robustness. This alignment further supports the utility of tertile categorisation for risk stratification in clinical practice.

The results of the incremental analysis in this study indicate that LAR and NT-proBNP have complementary value in risk stratification: NT-proBNP is primarily used to identify high-risk patients, whilst LAR excels at identifying patients with a favourable actual prognosis from the high-risk cohort identified by NT-proBNP (NRI- significant improvement). This characteristic makes LAR an ideal “exclusion” marker, with its core value lying in reducing false positives and avoiding unnecessary intensive interventions. Based on this, and in conjunction with LAR's tertile grouping (LAR ≤ 4.30, LAR2 4.30–5.53, LAR3 > 5.53), we propose a hypothetical clinical management framework. LAR3 (>5.53): High-risk patients; increased follow-up frequency and initiation of intensive treatment assessment are recommended; LAR2 (4.30–5.53): Moderate-risk patients; routine follow-up is recommended; LAR1 (≤4.30): Low-risk patients; a reduction in the frequency of follow-up may be considered; This stratification approach translates the statistical added value of LAR into a reference for clinical risk management, thereby helping to optimise the allocation of healthcare resources.

However, several limitations should be acknowledged. First, as a single-centre retrospective study, it may be subject to selection bias; further validation is required through multicentre prospective studies. Second, serum LDH testing lacks specificity, and the LAR may reflect the overall severity of the disease rather than pathological processes specific to heart failure. Additionally, this study did not include data on HF etiology (ischaemic vs. non-ischaemic), device therapy (ICD/CRT) or other relevant inflammatory markers. When interpreting the results of this study, the aforementioned limitations should be taken fully into account. Future prospective multicentre studies incorporating additional confounders are needed to further validate our findings.

In summary, this study demonstrates that baseline LAR levels serve as independent predictors of HF-related readmission, all-cause mortality, and composite endpoints in both HFrEF and HFmrEF patients, highlighting their significant value in clinical risk stratification.

## Data Availability

The raw data supporting the conclusions of this article will be made available by the authors, without undue reservation.
