# Supplementary material for: Lactate dehydrogenase-to-albumin ratio and adverse outcomes in patients with HFrEF and HFmrEF
Source: Front Cardiovasc Med. 2026 Apr 27;13:1786253. doi: 10.3389/fcvm.2026.1786253 (PMC13158801; doi:10.3389/fcvm.2026.1786253)
Supplement: Supplementary file 1 [file Table1.docx]

Supplementary Table 1

Results of the Cox proportional hazards hypothesis test (Schoenfeld residuals method)

| Endpoint | Model | **Global test** | | | **LAR tertile** | **Variables violating assumptions** |
| --- | --- | --- | --- | --- | --- | --- |
|  |  | χ² | df | *P* | *P* |  |
| HF-related  readmission | Model1 | 1.06 | 2 | 0.590 | 0.775 | None |
|  | Model2 | 4.86 | 7 | 0.676 | 0.886 | None |
|  | Model3 | 21.46 | 25 | 0.667 | 0.775 | None |
| All-cause mortality | Model1 | 1.71 | 2 | 0.426 | 0.585 | None |
|  | Model2 | 2.33 | 6 | 0.887 | 0.755 | None |
|  | Model3 | 15.55 | 24 | 0.904 | 0.585 | None |
| Composite endpoint | Model1 | 3.29 | 2 | 0.193 | 0.193 | None |
|  | Model2 | 8.44 | 8 | 0.392 | 0.258 | None* |
|  | Model3 | 27.75 | 27 | 0.424 | 0.378 | None |

*Note: In Model 2 with a composite endpoint, the p-value for AF was 0.044, which is slightly below 0.05; however, in the fully adjusted Model 3, the p-value rose to 0.162, and all global test p-values were greater than 0.05, indicating that the overall model satisfies the proportional hazards assumption.
