# Supplementary material for: Lactate dehydrogenase-to-albumin ratio and adverse outcomes in patients with HFrEF and HFmrEF
Source: Front Cardiovasc Med. 2026 Apr 27;13:1786253. doi: 10.3389/fcvm.2026.1786253 (PMC13158801; doi:10.3389/fcvm.2026.1786253)
Supplement: Supplementary file 2 [file Table2.docx]

|  | **Variable count** | **VIF Range** | **VIFmax** | **<10** |
| --- | --- | --- | --- | --- |
| HF-related readmission | 24 | 1.06–7.12 | 7.12 | yes |
| All-cause mortality | 23 | 1.07–6.93 | 6.93 | yes |
| Composite endpoint | 26 | 1.08–7.19 | 7.19 | yes |

Supplementary Table 2 Testing for Multicollinearity in Endpoint Events
