# Supplementary material for: Lactate dehydrogenase-to-albumin ratio and adverse outcomes in patients with HFrEF and HFmrEF
Source: Front Cardiovasc Med. 2026 Apr 27;13:1786253. doi: 10.3389/fcvm.2026.1786253 (PMC13158801; doi:10.3389/fcvm.2026.1786253)
Supplement: Supplementary file 3 [file Table3.docx]

| Variable | HF-related readmission | | | All-cause mortality | | | Composite endpoint | | |
| --- | --- | --- | --- | --- | --- | --- | --- | --- | --- |
|  | HR | 95%CI | *P* | HR | 95%CI | *P* | HR | 95%CI | *P* |
| Age | 1.023 | 1.011-1.035 | ＜0.001 | 1.047 | 1.030-1.064 | ＜0.001 | 1.031 | 1.021-1.041 | ＜0.001 |
| Gender | 1.475 | 1.111-1.959 | 0.007 | 1.221 | 0.834-1.789 | 0.305 | 1.309 | 1.029-1.664 | 0.028 |
| Admission systolic blood pressure | 0.991 | 0.985-0.997 | 0.003 | 0.995 | 0.987-1.003 | 0.206 | 0.993 | 0.988-0.998 | 0.007 |
| Admission diastolic blood pressure | 0.987 | 0.978-0.995 | 0.002 | 0.990 | 0.980-1.001 | 0.086 | 0.990 | 0.983-0.997 | 0.003 |
| BMI | 1.001 | 0.995-1.007 | 0.761 | 0.981 | 0.937-1.027 | 0.409 | 1.000 | 0.994-1.006 | 0.996 |
| History of hypertension | 0.949 | 0.735-1.225 | 0.686 | 0.959 | 0.688-1.336 | 0.806 | 0.994 | 0.806-1.226 | 0.954 |
| History of diabetes | 1.019 | 0.757-1.372 | 0.902 | 1.111 | 0.761-1.621 | 0.587 | 1.068 | 0.838-1.361 | 0.594 |
| Previous history of PCI | 0.653 | 0.494-0.863 | 0.003 | 0.653 | 0.494-0.863 | 0.069 | 0.659 | 0.524-0.830 | ＜0.001 |
| History of stroke | 1.124 | 0.655-1.929 | 0.671 | 2.074 | 1.214-3.542 | 0.008 | 1.549 | 1.053-2.280 | 0.026 |
| Combined with AF/AFL | 2.270 | 1.738-2.965 | ＜0.001 | 2.123 | 1.507-2.992 | ＜0.001 | 2.235 | 1.793-2.787 | ＜0.001 |
| Combined with ventricular arrhythmia | 1.481 | 1.133-1.938 | 0.004 | 1.636 | 1.163-2.301 | 0.005 | 1.563 | 1.255-1.947 | ＜0.001 |
| NYHA |  |  | ＜0.001 |  |  | ＜0.001 |  |  | ＜0.001 |
| Class II |  |  | Ref. |  |  | Ref. |  |  | Ref. |
| Class III | 1.800 | 1.310-2.474 | ＜0.001 | 2.732 | 1.730-4.314 | ＜0.001 | 2.014 | 1.540-2.633 | ＜0.001 |
| Class IV | 1.796 | 1.732-3.535 | ＜0.001 | 3.334 | 2.052-5.417 | ＜0.001 | 2.783 | 2.092-3.701 | ＜0.001 |
| WBC | 0.991 | 0.931-1.056 | 0.783 | 1.074 | 1.003-1.151 | 0.041 | 1.039 | 0.991-1.090 | 0.114 |
| HGB | 0.991 | 0.985-0.997 | 0.002 | 0.985 | 0.977-0.992 | ＜0.001 | 0.990 | 0.985-0.995 | ＜0.001 |
| PLT | 0.999 | 0.997-1.001 | 0.150 | 0.997 | 0.994-0.999 | 0.015 | 0.998 | 0.996-1.000 | 0.011 |
| GLU | 1.017 | 0.985-1.051 | 0.303 | 1.015 | 0.973-1.058 | 0.503 | 1.020 | 0.993-1.047 | 0.147 |
| ALB | 0.917 | 0.893-0.943 | ＜0.001 | 0.880 | 0.851-0.909 | ＜0.001 | 0.905 | 0.885-0.925 | ＜0.001 |
| LDH | 1.003 | 1.002-1.004 | ＜0.001 | 1.003 | 1.002-1.004 | ＜0.001 | 1.119 | 1.093-1.145 | ＜0.001 |
| CR | 1.008 | 1.003-1.012 | 0.001 | 1.014 | 1.010-1.019 | ＜0.001 | 1.011 | 1.008-1.014 | ＜0.001 |

Supplementary Table 3: Univariate Cox Regression Analysis of the Endpoint Event

| Variable | HF-related readmission | | | All-cause mortality | | | Composite endpoint | | |
| --- | --- | --- | --- | --- | --- | --- | --- | --- | --- |
|  | HR | 95% CI | *P* | HR | 95%CI | *P* | HR | 95%CI | *P* |
| eGFR | 0.986 | 0.980-0.993 | ＜0.001 | 0.975 | 0.968-0.982 | ＜0.001 | 0.981 | 0.976-0.986 | ＜0.001 |
| Blood sodium | 0.975 | 0.964-0.986 | ＜0.001 | 0.985 | 0.970-0.999 | 0.038 | 0.976 | 0.967-0.985 | ＜0.001 |
| Blood potassiu | 0.846 | 0.633-1.131 | 0.258 | 1.058 | 0.728-1.537 | 0.768 | 0.924 | 0.727-1.175 | 0.518 |
| UA | 1.001 | 1.001-1.001 | ＜0.001 | 1.001 | 1.001-1.001 | ＜0.001 | 1.001 | 1.001-1.001 | ＜0.001 |
| HCY | 0.996 | 0.988-1.005 | 0.400 | 1.011 | 1.004-1.017 | 0.002 | 1.004 | 0.998-1.009 | 0.197 |
| TNI | 1.363 | 1.065-1.745 | 0.014 | 1.586 | 1.322-1.902 | ＜0.001 | 1.510 | 1.298-1.757 | ＜0.001 |
| NT-proBNP | 1.000 | 1.000-1.000 | ＜0.001 | 1.000 | 1.000-1.000 | ＜0.001 | 1.000 | 1.000-1.000 | ＜0.001 |
| D-dimer | 1.031 | 1.004-1.060 | 0.027 | 1.074 | 1.003-1.151 | ＜0.001 | 1.039 | 1.019-1.060 | ＜0.001 |
| LAD | 1.631 | 1.445-1.841 | ＜0.001 | 1.504 | 1.261-1.793 | ＜0.001 | 1.569 | 1.412-1.742 | ＜0.001 |
| LVLDs | 1.149 | 1.006-1.312 | 0.040 | 1.063 | 0.897-1.260 | 0.479 | 1.117 | 1.002-1.246 | 0.047 |
| LVLDd | 1.133 | 0.982-1.307 | 0.087 | 1.076 | 0.897-1.291 | 0.431 | 1.116 | 0.992-1.256 | 0.067 |
| RVAPD | 2.167 | 1.672-2.807 | ＜0.001 | 2.488 | 1.812-3.418 | ＜0.001 | 2.303 | 1.869-2.838 | ＜0.001 |
| LVEF | 0.951 | 0.935-0.968 | ＜0.001 | 0.953 | 0.932-0.974 | ＜0.001 | 0.952 | 0.939-0.966 | ＜0.001 |
| Beta-blockers | 1.028 | 0.663-1.593 | 0.903 | 0.732 | 0.446-1.200 | 0.216 | 0.888 | 0.632-1.249 | 0.496 |
| ACEI/ARB/ARNI | 1.364 | 0.880-2.115 | 0.165 | 0.689 | 0.441-1.076 | 0.102 | 1.047 | 0.756-1.451 | 0.780 |
| MRA | 2.719 | 1.899-3.891 | ＜0.001 | 2.091 | 1.348-3.244 | 0.001 | 2.454 | 1.845-3.266 | ＜0.001 |
| SGLT2i | 1.602 | 1.204-2.132 | 0.001 | 1.450 | 1.002-2.096 | 0.049 | 1.552 | 1.227-1.962 | ＜0.001 |
| Anticoagulants | 2.267 | 1.745-2.944 | ＜0.001 | 2.067 | 1.473-2.900 | ＜0.001 | 2.188 | 1.762-2.717 | ＜0.001 |
| LAR Group |  |  |  |  |  |  |  |  |  |
| LAR1 |  |  | Ref. |  |  | Ref. |  |  | Ref. |
| LAR2 | 1.533 | 1.069-2.199 | 0.020 | 2.260 | 1.312-3.894 | 0.003 | 1.697 | 1.247-2.309 | 0.001 |
| LAR3 | 3.080 | 2.212-4.288 | ＜0.001 | 4.843 | 2.940-7.977 | ＜0.001 | 3.562 | 2.687-4.722 | ＜0.001 |
| *P* for trend |  |  | ＜0.001 |  |  | ＜0.001 |  |  | ＜0.001 |

Note: Same as Table 1.
