# Supplementary material for: Lactate dehydrogenase-to-albumin ratio and adverse outcomes in patients with HFrEF and HFmrEF
Source: Front Cardiovasc Med. 2026 Apr 27;13:1786253. doi: 10.3389/fcvm.2026.1786253 (PMC13158801; doi:10.3389/fcvm.2026.1786253)
Supplement: Supplementary file 4 [file Table4.docx]

Supplementary Table 4. Comparison of the predictive value of LAR and NT-proBNP (24 months)

| Endpoint | Model | C-index(95%CI) | ΔC-index | P | NRI (95% CI) | P | NRI+ (95% CI) | NRI- (95% CI) | IDI (95% CI) | P |
| --- | --- | --- | --- | --- | --- | --- | --- | --- | --- | --- |
| HF-relatd readmission | Model 1 | 0.664 (0.628-0.701) | **-** | **-** | **-** | **-** | **-** | **-** | **-** | **-** |
|  | +NT-proBNP | 0.684 (0.650-0.719) | +0.020 | **0.007** | ****0.227 (0.064-0.393)**** | ****0.007**** | ****0.158(0.036-0.303)**** | 0.069 (-0.004-0.126) | 0.0122(-0.022-0.046) | 0.490 |
|  | +LAR | 0.689 (0.655-0.723) | +0.005 | 0.083 | 0.171(-0.027-0.381) | 0.075 | -0.097(-0.193-0.083) | ****0.268 (0.114-0.369)**** | 0.0056(-0.014-0.025) | 0.570 |
| All-cause mortality | Model 1 | 0.739 (0.698-0.779) | **-** | **-** | **-** | **-** | **-** | **-** | **-** | **-** |
|  | +NT-proBNP | 0.780 (0.744-0.815) | ****+0.041**** | 0.003 | 0.426(0.228-0.650) | ****0.004**** | ****0.232 (0.099-0.397)**** | 0.193 (0.093-0.278) | ****0.0319(0.0058-0.062)**** | 0.464 |
|  | +LAR | 0.781 (0.745-0.816) | +0.001 | 0.733 | 0.217(-0.116-0.386) | 0.131 | -0.089(-0.292-0.087) | 0.307 (-0.005-0.422) | 0.0055(-0.0021-0.0142) | 0.482 |
| Composite endpoints | Model 1 | 0.689 (0.660-0.718) | **-** | **-** | **-** | **-** | **-** | **-** | **-** | **-** |
|  | +NT-proBNP | 0.717 (0.690-0.745) | ****+0.029**** | 0.0001 | ****0.305 (0.181-0.463)**** | ****0.004**** | ****0.176 (0.083-0.292)**** | 0.129 (0.066-0.200) | **0.0255(0.086-0.0409)** | 0.500 |
|  | +LAR | 0.721 (0.695-0.748) | ****+0.004**** | 0.097 | 0.167(-0.037-0.333) | 0.131 | -0.084(-0.193-0.036) | ****0.251 (0.112-0.348)**** | 0.0006(-0.0062-0.0063) | 0.870 |

Note: Model 1: age, sex, NYHA functional class, LVEF, eGFR, log(TnI + 0.001);

NRI: Net Risk Improvement; NRI+: net risk improvement in the event group; NRI–: net risk improvement in the non-event group；

Bold text indicates that the 95% CI does not cross zero, indicating statistical significance.
